# Exosome-Based Nanoplatforms: The Emerging Tools for Breast Cancer Therapy

**DOI:** 10.3389/fonc.2022.898605

**Published:** 2022-04-28

**Authors:** Quan Liu, Xian Zhang, Jun Zhang

**Affiliations:** ^1^ Department of Thyroid and Breast Surgery, Xiantao First People’s Hospital Affiliated to Yangtze University, Xiantao, China; ^2^ Ningxia Key Laboratory of Cerebrocranial Diseases, Incubation Base of the National Key Laboratory, Ningxia Medical University, Yinchuan, China; ^3^ Department of Thyroid and Breast Surgery, Shenzhen Qianhai Shekou Free Trade Zone Hospital, Shenzhen, China

**Keywords:** breast cancer, exosome, nanoparticle, therapy, drug-resistance

## Abstract

Breast cancer (BC) remains the leading malignant tumor type among females worldwide. The patients with BC are still faced with undesirable metastasis, relapse rate, and drug resistance. Exosomes are defined as naturally occurring extracellular vesicles (EVs) with typical biomarkers that reflect the characteristics of the parent cells. Exosomes are crucial mediators involved in intercellular communication. By transferring multiple cargoes, represented by proteins, nucleic acids, lipids, metabolites, exosomes contribute to reshaping the recipient cell function and fate. Growing evidence has documented that exosomes originating from BC cells are important participants involved in BC progression and treatments. Nanoparticle-based technology is the cutting-edge field for renewing pharmaceuticals and has endowed deep improvements in precise BC treatment. Additionally, due to their perfect features of the low immune prototype, limited adverse effects, prolongated circulation, and easy modification, exosomes have received much attention as candidates in nano-medicine of BC. The nanoplatforms constructed by exosomes have safety, intelligence, biomimetic, and controlled released advantages for combating BC. Here, we emphasize the multiple exosomes from a variety of cell sources in constructing nanoplatforms for BC therapy, mainly including exosomes and their cargoes, genetically engineered exosomes, and exosome-based carriers. This field would shed light on the promising exosome-based delivery system in BC therapy.

## Introduction

Breast cancer (BC) remains the leading cause of cancer-caused death in females and its incidence is still rising worldwide (1). Despite the advancement of early diagnosis procedures and mature therapeutic strategies, such as mastectomy, chemotherapy, immunotherapy, and combined therapy, BC patients are still faced with vicious metastasis, relapse rate, and drug resistance (2). BC is a highly heterogeneous and complex entity. The BC tumor microenvironment (TME) consists of tumor cells and stromal cells, soluble cytokines, immune cells, which directly or indirectly impact tumor behaviors and thus establish tumor-favoring niches for supporting tumorous growth and colonization (3). It is still a difficult problem to figure out the detailed mechanism of tumor occurrence and development and improve the efficiency of tumor treatment strategies.

Exosomes are defined as naturally occurring extracellular vesicles (EVs) with approximately 30-150 nm in diameter (4). Exosomes are originated from endocytic multivesicular bodies (MVBs) followed by exosome formation and secretion into extracellular space. In respect of the typical biomarkers, exosomes are particularly rich in a series of conserved proteins that reflect the characteristics of the parent cells, including the tetraspanin transmembrane-4 family (CD9, CD63, and CD81), Hsp90, caveolins, clathrin, and transferrin receptors (5). Generally speaking, exosomes serve as a warehouse that stores a large amount of biologically active molecules, such as lipids, enzymes, mRNAs, metabolites, and various non-coding RNAs, represented by miRNAs, long noncoding RNAs (lncRNAs), and circular RNA (circRNAs) (6). By transferring these cargoes, exosomes play roles in dynamic intercellular communication between tumor cells and adjacent stromal compartments in cancer progression (7). It is well-documented that tumor-derived exosomal RNAs affect the function of recipient cells in the neighborhood and distant sites, leading to tumor growth, metastasis, detection, and drug resistance in BC (8). For example, Ohno et al. used exosomes to deliver let-7a miRNA, which was transferred and internalized to epidermal growth factor receptor (EGFR)-expressing in tumor tissues, exhibiting extraordinary tumor therapeutic effects (9). Santos et al. reported that miR-155 in exosomes isolated from cancer stem cells (CSCs) and resistant cells could be transferred to recipient cells (10). This implies that exosomes may modulate resistance and migration ability to recipient BC cells partially through exosomal transfer to sensitive tumor cells.

Nanoparticle-based technology has endowed deep improvements in precise BC treatment. In consideration of the excellent biosafety, low immunogenicity, carrier properties, and nanoscale penetration effect, exosomes have attracted considerable attention in drug delivery systems for cancer therapy (11). The reported applications of exosomes mainly vary from enhanced efficiency in cancer drug delivery, drug-carrying system, to immunogenicity of cancer vaccines (12). The nanoplatforms constructed by exosomes possess enhanced functionalities with safety, intelligence, biomimetic, and controlled released advantages for combating BC (13). Therefore, this review aims to decipher the exosome potential serving as therapeutic carriers, hoping for offering an in-depth understanding of exosome-based based nanoplatforms for BC therapy.

To summarize the landscape of the potential applications, we have searched exosomes, breast cancer, and therapy on Pubmed over the last 10 years as keywords. These pieces of topically-relevant literature, involving molecular, cellular, and animal studies as well as clinical samples, are all included in this review.

## Exosomes and Their Cargoes

### Exosomes in BC Therapy

Mesenchymal stem cells (MSCs) have gained increasing interest in the field of regeneration and disease treatment due to their multilineage differentiation potential and powerful immunomodulatory and regenerative functions (14). MSC-derived exosomes are a new cell-free alternative to MSCs that has long been a major concern (15). MSC-derived exosomes offer unparalleled advantages in terms of safety, bioactivity, storage, and transport compared to previous MSC transplantation (16). Yu et al. posed that miR-342-3p was down-expressed in advanced BC patients and was of potential to suppress BC metastasis, cell survivability, and drug resistance (17). This result was partially caused by MSC-derived exosome miR-342-3p through binding ID4. The clinical samples with primary BC showed poor expression of miR-148b-3p. In both *in vitro* and *in vitro* validation, human umbilical cord mesenchymal stem cells (HUCMSCs) -derived exosomes carrying miR-148b-3p showed efficient inhibition of MDA-MB-231 cells (18). This suggested that miR-148b-3p-containing exosomes might represent an efficient and facile carrier for BC treatment. In addition, HUCMSC exosomes harboring miR-3182 could inhibit triple-negative breast cancer (TNBC) in invasion *in vitro*, demonstrating that miR-3182-containing exosomes may be a dependable therapeutic option in treating TNBC (19).

Natural killer (NK) cells are intrinsic lymphocytes that play a key role in tumor immune surveillance and are being actively investigated for adoptive cell therapies in cancer immunotherapy (20). NK cell-derived exosomes have a natural and excellent killing effect on tumor cells. The canine NK-exosomes were capable of significantly suppressing tumor size and reducing CD133 expression, representing a promising vehicle for the treatment in an experimental murine BC model (21). Zhu et al. separated the exosome mimetics (NK-EM) from NK cells by extruding NK cells into filters with gradually smaller pore sizes (22). NK-EM exhibited strong tumor-killing activity against tumors in mice such as glioblastoma and BC, compared to low or high doses of NK-Exo.

### Exosome Pre-Condition in BC Therapy

Several studies have shown that the use of exosome derivatives or exosome pre-injection can reduce the accumulation of other exosomal carriers or nanodrug carriers in the liver and thus more effectively promote drug enrichment at the target site. Based on exosomes derived from metastatic 4T1 cells, exosome-like nanovesicles (ENVs) were developed (23). Pre-treatment with 4T1 ENVs reduced the evasion of Kupffer cell-mediated phagocytosis by DOTAP/DOPE liposomes, thereby promoting greater recruitments of DOTAP/DOPE carriers to tumor metastasis, reducing the IC50 of chemotherapeutic drug doxorubicin (DOX), and avoiding adverse side effects. Besides, the accumulation of subsequently injected grapefruit-derived nanovector (GNV) decreased in the lungs, but not the liver, was increased by prior intravenous injection of peripheral blood-derived exosomes in mice (24). Meanwhile, the efficiency of GNV-carrying DOX or paclitaxel (PTX) for the treatment of lung metastases was improved. Exosome-mediated inhibitory effects of GNV into hepatic macrophages were dependent on CD36 and IGFR1 receptor-mediated pathways. Thus, autologous exosome pre-treatment not only accumulated GNV signals in the primary tumor, but also improved the therapeutic efficacy against pulmonary metastases. Melzer et al. treated MSC with sublethal concentrations of PTX and found that the treated MSC exosomes had more potent tumor-specific and targeting properties compared with PTX of equivalent effect (25). In addition, the distribution of MSC PTX exosomes in major organs was reduced by approximately 50% and effectively reduced subcutaneous graft tumor volume by 60%.

## Genetically Engineered Exosomes

Several features of MSCs may also be transmitted to exosomes, including weak immunogenicity with lack of MHC-II and co-stimulatory ligands, multi-organ homing ability mediated by the expression of specific surface molecules, and high biosafety characteristics (26). The available reports are controversial as to whether MSC-derived exosomes are tumor-promoting or tumor-suppressing in different tumor types. However, MSC-derived exosomes modified by gene editing can certainly overexpress a specific therapeutic RNA or protein to exert tumor-suppressive effects.

MSC exosomes expressing suicide or tumor-killing genes represent an emerging class of tumor-targeting drugs and vector models that act within tumor cells for the treatment of breast tumors (27). Exosomes from generationally modified MSCs can perform as an effective targeting delivery system to specifically target HER2+ cell lines, resulting in corresponding changes in tumor death (28). O’Brien et al. harnessed the tumor homing ability of MSCs to construct miR-379-enriched MSC-EVs, which could significantly inhibit BC tumor growth and promote necrosis, depending on the regulation of cyclooxygenase-2 (COX-2) expression (29). Vakhshiteh et al. established an ex-novo exosome nanocarrier, which was derived from miR-34a-overexpressed dental pulp mesenchymal stem cells (DPSCs), weakened the capability of migration and invasion in MDA-MB-231 cells (30). MSCs-Exo effectively transported LNA-antimiR-142-3p to breast CSC-like cells, thereby reducing miR-142-3p and miR-150 expression (31). Furthermore, inhibiting oncomiRs by transmitting LNA-antimiR-142-3p induced a dramatic lowering of clone formation and tumor initiation capability.

Genetically engineered T cells expressing chimeric antigen receptors (CARs) have become a force to be reckoned with in tumor immunotherapy (32). Exosomes derived from CAR-T cells could facilitate BC immunotherapy by providing higher efficacy and safety (33). Yang et al. successfully isolated exosomes from mesothelin (MSLN)-targeted CAR-T cells, which maintained most of the traits of their parental T cells, including surface expression of CARs and CD3 (34). In addition, CAR-carrying exosomes significantly suppressed the growth of MSLN-positive TNBC cells by secreting perforin and granzyme B, as well as efficiently inhibited the xenograft tumors without noticeable side effects. Conversion of M2 macrophages to the M1 phenotype employing miRNA-containing exosomes is a possible route to inhibit BC tumor invasion and metastasis (35). By using tumor-derived exosomes as carriers, miR-130 was transported to M2 macrophages, which in turn impaired the ability of tumor cells to proliferate, migrate and invade (36). Yue et al. reported that PGRN-/- TAMs restrained BC cell invasion and epithelial to mesenchymal transition (EMT) through releasing their exosomes (37). MiR-5100 upregulation of PGRN-/- TAMs-derived exosomes might modify CXCL12 expression, thereby dampening the CXCL12/CXCR4 axis and consequently resulting in BC malignant alteration. Shi et al. reported the genetically engineered cell-derived platform for developing targeted BC immunotherapy (38). They constructed a dual-targeting exosome platform for T-cell CD3 and BC-specific human epidermal growth factor receptor 2 (HER2) receptors, which was capable of targeted activation of CD8+ T cells and potent killing of HER2 tumor cells, demonstrating promising immunotherapeutic effects.

Adipose-derived stem cells (ADSCs) are considered to be an important tool for cell therapy and regeneration because of their abundant source, easy extraction, and ability to multidirectional differentiation (39). Exosomes are an important bearer form of the secretory profile of ADSCs. Exosomes are capable of delivering to recipient cells some nucleic acids, small molecules, and protein substances that are embedded in MSCs (40). Shojaei et al. successfully isolated ADSC-exosomes and found that miR-381 mimics were efficiently conveyed to MDA-MB-231 cells by ADSC-exosomes (41). Remarkably, ADSC-exosomes loaded with miR-381 inhibited the growth and metastasis ability of MDA-MB-231 cells and promoted apoptosis *in vitro*, showing the excellent RNA therapy potential of ADSC-exosomes. Sheykhhasan et al. constructed miR-145-overexpressing exosomes from ADSCs *via* lentivirus vector (42). These exosomes significantly inhibited tumor cell growth and metastasis by delivering miR-145.

In addition, the study of Li et al. generated the exosomes containing siMTA1 by using the electroporation method, which increased the gemcitabine-mediated inhibition of tumor growth *in vivo* by reversing the EMT effect and inhibiting the autophagic process (43). Limoni et al. transduced HEK293T cells with a lentiviral vector bearing LAMP2B-DARPin G3 chimeric gene for targeting HER2-specific tumors (44). Then, the exosomes generated from these cells were isolated and then loaded with TPD52-silencing siRNAs, which were subsequently delivered to SKBR3 cells for reducing tumor. Hu et al. constructed exosome-like nanovesicles (eNVs-FAP) from fibroblast activation protein-α (FAP) gene-engineered tumor cells (45). These nanovesicles facilitated dendritic cell (DC) mature, T cell, and FAP+CAFs infiltration, and depressed the ratio of immunosuppressive M2, myeloid-derived suppressor cells (MDSCs), and regulatory T cells (Tregs). Therefore, it implied that eNVs-FAP was a promising oncologic vaccine for targeting both the parenchyma and the stroma of BC tumors.

## Exosome-Based Carrier Construction

### Exosomes Serve as Targeted Coating Substances

The use of exosomes to carry conventional clinical chemotherapy drugs can effectively reduce the toxicity of the drugs and improve the enrichment effect of targeted sites (46). Exploiting the targeting capability of BC cell exosomes can empower the homing ability of the nanoplatforms to target homologous tumor cells. Therefore, exosomes of tumor cells or other functional cells can serve as targeted coating substances in nanoplatforms. For example, Chen et al. prepared the smart bionanodrug EXO-GO-CO-γ-PGA-MIT for delivering mitoxantrone (MIT) by exploiting the targeting properties of BC cell-originated exosomes (47). This nanodrug possesses slow-release, tumor aggregation, and enhanced pro-tumor apoptotic ability of MIT. Ghavami et al. established a radiolabeled exosome-based tracer [111In]In-oxine-T-exos, which was markedly internalized by HER2-positive cells for imaging HER2-expressing tumor (48).

Interestingly, a bioactivated exosome-based nanoplatform (EMPCs), was formulated by reactive oxygen species (ROS)-reactive thioether-linked paclitaxel-linoleic acid conjugates (PTX-S-LA), and cucurbitacin B (CuB) was co-loaded in polymeric micelles, with exosome-decorated membranes (49). EMPCs not only displayed enlarged prodrug function, increased blood circulation, the targeted capability of homozygous tumor cells, and improved tumor penetration, but also inhibited BC metastasis through circulating tumor cells (CTCs) elimination and FAK/MMP pathway modulation. Li et al. developed an engineered macrophage exosome to encapsulate poly(lactic-co-glycolic acid) nanoparticles and modified with c-Met-targeting peptide on the exosome surface for tumor targeting (50). This exosome nanocomplex (MEP-D), exhibited significant tumor targeting and tumor killing functions. Zhao et al. designed the CBSA/siS100A4@Exosome by conjugating cationic bovine serum albumin (CBSA) and siS100A4 followed with exosome membrane encapsulation (51). CBSA/siS100A4@Exosome possessed a higher binding affinity to the lung and superiorly exhibited metastasis-associated protein S100A4 expression, showing great inhibition potential in malignant BC growth and lung metastasis. ID@E-MSNs was a tumor cell-derived exosome-mimetic porous silica nanoparticles as an integrated drug delivery platform for carrying both indocyanine green (ICG) and DOX (52). In 4T1 tumor-bearing mice, the nanoparticles were able to enrich at the tumor site and promote ICG thermal effect-induced drug release and tumor ablation under 808 nm NIR irradiation, thus enabling combined chemotherapeutic BC treatment.

### Exosomes Serve as Drug Delivery Carriers

The carrier properties of exosomes can be directly used to load chemotherapeutic drugs, photosensitizers or antitumor drugs. Exosomes are natural nanocarriers that can target the cancer-sensitizing agent indocyanine green (ICG) in a biosafe manner. FA-ExoICG was an engineered exosome that possessed tumor-targeting ligand folic acid (FA) and internally loaded ICG (53). Significant inhibition of MCF-7 tumor growth in mice was observed by a single intravenous injection of FA-ExoICG with followed ultrasound (US) irradiation, with a favorable biosafety profile. Tran et al. innovatively used a one-step strategy of loading amorphous nanomatrix formation into exosomes, such as encapsulation of aspirin into exosomes, which could effectively improve the efficiency of drug dissolution and homing targeting effect (54). This compound displayed toxic and killing effects on BC cells and colon cancer cells. Yu et al. developed a nano-carrier Erastin@FA-exo containing erastin-loaded exosomes labeled with FA (55). The results showed that Erastin@FA-exo targeted and inhibited the proliferation and migration of MDA-MB-231 cells and promoted the depletion of intracellular glutathione and activation of ROS to induce ferroptosis. Kalimuthu et al. synthesized personalized exosome mimics (EMs) loaded with PTX, termed PTX-MSC-Ems (56). The exosome carrier significantly inhibited the growth and tumor progression of MDA-MB-231 cells, and was regarded as a powerful drug delivery carrier for BC. Li et al. incorporated Dox in milk exosomes (mExo) and modified with specifically CD44-targeting hyaluronic acid (HA), named HA-mExo-Dox (57). This vector effectively targeted CD44-expressed BC cells and induced cell death *in vitro*. Gong et al. generated a biomimetic delivery system using A15-Exo to package cholesterol-modified miR-159 and chemotherapeutic agent DOX (58). *In vivo*, this delivery system effectively reduced the TCF-7 gene and exhibited potent anti-cancer effects without side effects. Thus, this study demonstrated the synergistic effect of exosomal transport carriers for the co-delivery of gene drugs and chemotherapeutic agents in TNBC treatment.

Immune cell-derived exosomes have parental cell properties and can be used to mimic immune cell targeting of cancer (59). By hybridizing sEV from mouse macrophages with synthetic liposomes, vesicles smaller than 200 nm in size can be designed to mimic the size of exosomes, denoting as hybrid exosomes (HE). The established HE maintained the good property of small EVs (sEVs) with higher colloidal stability, drug carrier feature, and durable release of DOX in response to pH, for killing 4T1 cells. Tian et al. established a well-characterized tool of exosome carrier by an αv integrin-specific iRGD peptide to mouse immature dendritic cells (imDCs) (60). These iRGD-expressing imDCs actively targeted BC tissues with high expression of integrin receptors, leading to significant tumor growth inhibition with limited systemic toxicity.

## Discussion

Due to the heterogeneity and biological barriers of tumors, conventional drugs for combating tumors are often difficult to achieve the balance between optimal drug effectiveness and minimum side effect. The majority of anti-cancer agents in clinical practice are toxic to cause adverse damage to normal cells with poor bioavailability, and insufficient *in vivo* stability. Nanoparticle-based technologies provide exciting approaches to BC diagnosis and therapeutics. Novel dosage forms represented by nanoplatforms are a cutting-edge way to improve the delivery efficiency of therapeutic agents. In addition to uploading drugs, the active targeting capability of nanoplatforms is also a factor that cannot be ignored. In terms of diagnosis, it is also worth mentioning that the exosome-related detection still has certain advantages, with non-invasive, high sensitivity and disease specificity, long circulation and stability. Specifically, exosome isolation methods mainly include ultracentrifugation techniques, polymer precipitation, size-based isolation, immunoaffinity chromatography, other isolation techniques. Each method has its own advantages and disadvantages. A more suitable separation method should be selected for different targets. In terms of identification, transmission electron microscopy (TEM) observation and nanoparticle tracking analysis (NTA) are commonly used to identify single exosomes with a diameter of 30-150 nm. Western blot detection can verify specific markers (such as CD63/CD81/CD9/TSG101/Alix), which can also be detected indirectly by immunofluorescence and flow cytometry (FCM). In addition, exosomes are a promising cell-free therapy, and the currently applied protection technologies mainly include freezing, freeze-drying, and spray-drying. In general, the isolation, extraction, identification and preservation technologies of exosomes are relatively mature, but the transformation and efficiency of exosomes are still relatively insufficient.

Currently, tumor targeting of nanoplatforms is mainly achieved *via* enhanced permeability and retention (EPR) effect in tumor internals or receptor-ligand interplay *via* adhering to overexpressed antigens on the tumor cell surface. In developing precisely targeting approaches, exosomes have emerged as ideal drug carriers due to their unique carrier properties and biosafety. In this review, exosome-related nanoplatforms are used for BC treatment, mainly including three aspects, exosomes and their cargoes, genetically engineered exosomes, and exosome-based carrier construction. Specifically, some cell-derived exosomes, or key components of exosomes themselves inhibit tumor proliferation. The genetically editing methods enable researchers to accurately manipulate the overexpression or knockdown of specific genes, thus facilitating the production of anti-tumor exosomes (**Table 1**). Notably, MSCs might secret a considerable amount of functionalized exosomes and are common gene-editing cell tools for yielding expressive-specific exosomes. In nanocarrier construction, surface modification of exosomes can increase specific target recognition and enhance recruitment and abundance at tumor sites. Encapsulation of nanomaterials by exosomes can prolong metabolic cycling, reduce clearance levels, and avoid drug degradation or inactivation. Exosomes can also be used as delivery platforms for carrying chemotherapeutics or other therapeutic agents such as phototherapy and photothermal therapy. Therefore, exosomes are also efficient multimodal synergistic vehicles for BC therapy. In addition, not only for treating BC, exosome carriers are also novel transport means investigated other tumors, including glioma, liver cancer, gastric cancer, and so on.

**Table 1:** The genetically engineered exosomes for combating BC.

Source of exosome	Carrier construction methods	Functions	Ref.
MSCs	an effective targeted delivery system by transduced MSCs	selectively targeted HER2+ cell lines	(28)
MSCs	engineered to secrete EVs enriched with miR-379	innovative therapy for metastatic breast cancer	(29)
DPSCs	miR-34a-overexpressing DPSCs were prepared using XMIRXpress-34a lentivectors	as a therapeutic carrier in BC cells *in vitro*	(30)
MSCs	a feasible nanovesicles to deliver RNA-based therapeutics	inhibited clone-formation and tumor-initiating abilities of the MCF7-derived cancer stem-like cells	(31)
CAR-T cells	MSLN-targeted CAR-T cells with MSLN-CAR construction	inhibited the growth of both endogenous and exogenous MSLN+ TNBC cells by perforin and granzyme B	(34)
4T1 cells	using miRNA-containing exosomes for therapeutic strategy	repolarization of M2 macrophages to M1 phenotype against tumor invasion and metastasis in breast cancer.	(36)
PGRN-/- TAMs	siPGRN-TAMs exosomes and PGRN-/- tumor tissue	inhibited invasion, migration, and EMT of breast cancer cells through their exosomes	(37)
Expi293 cells	developed an exosome platform termed synthetic multivalent antibodies retargeted exosome (SMART-Exo)	dually targeted T cell CD3 and HER2 receptors and exhibited highly potent and specific anti-tumor activity both *in vitro* and *in vivo*	(38)
ADSCs	Introduced miRNA-381-3p mimics to ADSC-exosomes by electroporation	inhibited proliferation, migration, and invasion capacity of MDA-MB-231 and promoted their apoptosis *in vitro*	(41)
MSCs	transfected with lentiviral vectors of miR-145-pLenti-III-enhanced green fluorescent protein (eGFP)	caused an improved anticancer property of miR-145 to inhibit BC	(42)
293T cells	Loading into exosomes of siMTA1 with electroporation.	increased the gemcitabine-mediated tumor growth inhibition effect *in vivo*	(43)
HEK293T cells	HEK293T cells were transduced by a lentiviral vector and seperated the exosomes to loaded with siRNA	bound specifically to HER2/Neu and were capable of delivering siRNA molecules against TPD52 gene into SKBR3 cells	(44)
Tumor cells	FAP gene-engineered tumor cell-derived exosome-like nanovesicles (eNVs-FAP) as a tumor vaccine	suppressed tumor growth by reprogramming TME and promoting tumor ferroptosis	(45)

Most studies suggest that tumor-associated exosomal components are a cancer-promoting factor. Reducing the release of tumor-associated or stromal cell exosomes also enhances the treatment efficacy. However, tumor-associated exosomal components can also act as important antigenic components to activate immune effects (61). At the same time, exosomes are capable to minimize the expression of drug resistance genes by delivering anti-miRNAs. Exosome-originated from MCF-7/ADR cells could promote active drug sequestration and induce drug resistance phenotypes by delivering resistance-related genes MDR-1 and P-glycoprotein (62). By reducing the resistant exosome formation and secretion, psoralen could reverse the development of drug resistance in BC cells. Also, there are some emerging novel exosome nanoplatform that have been developed for BC therapy. For instance, some novel T-cell-based vaccines are expected to perform the excellent tumor-killing function, by equipping polyclonal CD4+ T cells with antigen-specific exosomes (62). Exosomes derived from other cells, especially immune cells with tumor-killing effects, are also expected to be used as formulations for tumor therapy. *In vitro* studies have shown that tumor cells become more capable of activating T cells after DC-Exo uptake, thus potentially producing a more effective anti-tumor immune response, suggesting that DC-derived exosomes are also an effective exosome-related therapeutic tool (63). Or, combine with other treatment modes, exosomes as multifunctional carriers can be fully utilized. For example, heat stress increased the number of doxorubicin-containing exosomes in tumor cells and enhanced the antitumor effect of exosomes from doxorubicin-treated tumor cells (64). This suggests the potential for synergistic kill-expanding effects of combining chemotherapy and heat therapy for BC.

At present, although exosomes provide a variety of comprehensive and desirable properties for drug delivery, there are still many obstacles to be faced in this field. Firstly, there are many sources of cells currently used for exosome delivery, and it is hard to ensure the consistency and stability of the results of different research groups. The isolation and preparation of large amounts of engineered exosomes, including exosome purification, synthesis, stabilization, identification, and drug loading, remains a significant and complex step for BC tumor therapy. Secondly, when exosomes are coupled to nanoparticles or encapsulated with drugs, the metabolic kinetics of exosomes *in vivo* are worthy of further study. When circulating in the body, there may be a large number of liver retention or drug off-target phenomena, which will affect the efficiency of drug delivery. Finally, the current researches are preliminarily at the preclinical level, mostly at the level of cell and animal research. Due to the strict control of clinical trials and the complex nature of exosome components, exosome-based breast tumor therapy still has a long way to go. In order to finally achieve clinical application, it is necessary to carry out in-depth explorations on the preparation of exosome carriers, the real efficacy in the human body, and the control of side effects to determine the unification of the safety and effectiveness of exosome delivery.

## Conclusion

The naturally occurring exosomes, exosomes released by engineered or modified cells, exosomes that upload other substances, or exosomes that act as targeted coating substances, are several common forms of exosome-associated nanoplatforms for effective therapeutic carriers. Therefore, the comprehensive understanding of exosome biogenesis and the progress of efficient exosome engineering techniques will promote the clinical application of exosome-related drug nanoplatforms for combating BC.

## Author Contributions

All authors contributed to the design of the study and the writing of the manuscript. JZ designed the project and revised the manuscript. QL and XZ performed the literature search and wrote the manuscript. All authors reviewed the manuscript and approved the final version.

## Conflict of Interest

The authors declare that the research was conducted in the absence of any commercial or financial relationships that could be construed as a potential conflict of interest.

## Publisher’s Note

All claims expressed in this article are solely those of the authors and do not necessarily represent those of their affiliated organizations, or those of the publisher, the editors and the reviewers. Any product that may be evaluated in this article, or claim that may be made by its manufacturer, is not guaranteed or endorsed by the publisher.

## Abbreviations

ADSCs, adipose-derived stem cells; BC, breast cancer; CSCs, cancer stem cells; CARs, chimeric antigen receptors; circRNAs, circular RNA; CTCs, circulating tumor cells; CBSA, conjugating cationic bovine serum albumin; COX-2, cyclooxygenase-2; DPSCs, dental pulp mesenchymal stem cells; DOX, doxorubicin; EPR, enhanced permeability and retention; EGFR, epidermal growth factor receptor; EMT, epithelial to mesenchymal transition; NK-EM, exosome mimetics from NK cells; ENVs, exosome-like nanovesicles; eNVs-FAP, exosome-like nanovesicles; EVs, extracellular vesicles; FAP, fibroblast activation protein-α; FA, folic acid; GNV, grapefruit-derived nanovector; HER2,human epidermal growth factor receptor 2; HUCMSCs human umbilical cord mesenchymal stem cells; HA, hyaluronic acid; HE, hybrid exosomes; lncRNAs, long noncoding RNAs; MSCs, mesenchymal stem cells; MSLN, mesothelin; MIT, mitoxantrone; MVBs, multivesicular bodies (MVBs); MDSCs, myeloid-derived suppressor cells;NK, natural killer; PTX, paclitaxel;ROS, reactive oxygen species;Tregs, regulatory T cells; sEVs, small EVs; TNBC, Triple-negative breast cancer; TME, tumor microenvironment; US, ultrasound.
